# Caution warranted for low-dose radiation therapy for Covid-19

**DOI:** 10.1259/bjr.20200466

**Published:** 2020-10-28

**Authors:** Indra J Das, John A. Kalapurakal, Bharat B. Mittal

**Affiliations:** 1 Department of Radiation Oncology, Northwestern Memorial Hospital, Northwestern University Feinberg School of Medicine, Chicago, IL 60611, United States

## Abstract

Covid-19 is a morbid respiratory disease that has caused desperate times on a global scale due to the lack of any effective medical treatment. Some in the radiation community are actively proposing low-dose radiation therapy (LDRT) for managing the viral pneumonia associated with Covid-19. This commentary provides a rationale for exercising caution against such a decision as the efficacy of LDRT for viral diseases is unknown, while its long-term adverse risks are well known.

## Preface

The unprecedented conditions associated with Covid-19 has created an emergency of a magnitude that the world has not seen since the 1918 Spanish flu which killed nearly 50 million people worldwide. The Covid-19 virus is highly contagious, with a doubling time of 2–6 days. It is mainly a lung disease with a manifestation of pneumonia that could rapidly progress to multi-organ failure and strokes with fatal consequences. A number of underlying factors such as smoking history, diabetes, cancer, cardiac failure and other medical factors may result in an immunocompromised condition that may play an important role in determining the clinical outcome after infection. Covid-19 also has a high degree of morbidity depending on the country and the care that patients’ receive. Chest imaging (x-rays and CT scans) shows patchy or diffuse asymmetric airspace opacities with a distinct ground-glass pattern in the lungs. To date, there is no known medicine to cure this viral infection. Various clinical trials with many antiviral drugs are being proposed and conducted with limited success. In these desperate times, researchers in different medical specialties are trying to find ways to control the disease and provide various treatment options for these patients.^1^ Investigators in the radiation oncology community have proposed the use of low-dose (0.02–0.50 Gy) radiation treatment (LDRT) to manage the inflammation caused by Covid-19 pneumonia.^2,3^

### Hiding in the shadows of history

The proposed use of LDRT is actually a re-exploration of century-old anecdotal data on the efficacy of roentgen ray therapy. Primitive approaches for treatment with X-rays for every disease had been attempted without any scientific rationale. Heidenhain and Fried^4^ showed that roentgen rays can be used for benign conditions and inflammatory diseases, including viral pneumonia. In the USA, Park^5^ validated the work of Heidenhain and Fried in his MD thesis in 1938. The early days of therapeutic X-ray usage were hindered by the lack of knowledge of radiation risks as dosimetry and radiation biology were not fully developed and the mechanisms of radiation interactions with tissues were poorly understood.^6^ The anecdotal usage of X-rays for non-malignant conditions has waned with advances in medicine using antibacterial drugs. Even today, with all the advances in radiation dosimetry and biology, the effects of LDRT are still a mystery. This aspect is clearly demonstrated in the point/counterpoint article by Doss and Little.^7^

At the peak of the Covid-19 pandemic, the use of radiation treatments for cancer patients was restricted due to many factors including institutional lockdown, fears regarding viral exposure, lack of personal protective equipment, and the fear of unknowns. Recently, guidelines have been published for providing care to cancer patients during the pandemic, but these are institutional perspectives and may not be applicable globally.^8^ The use of LDRT could also be interpreted as a possible immune-response to radiation, which is gaining traction in immunotherapy but with traditional higher doses.^9^ Schröder et al^10^ have shown that LDRT in the range of 0.01–2.0 Gy is immunomodulatory and produces cytokine secretion of interlukin-8, granulocyte microphage colony stimulating factor, and platelet-derived growth factor. These effects of cytokines are both time- and dose-dependent as shown in the cell cultures. Similarly, with a 1–12 Gy dose, Rödel et al^11^ showed anti-inflammatory response with modulation of cytokines and activated microphages. These findings suggest that low doses may help in the management of pneumonia, but this could be an extrapolated view without the preclinical data to support its use.^12^

### Radiation risk

On the other hand, many believe that radiation risk has no threshold and is linear with dosage. Lessons learned from low-dose exposure associated with atomic bomb survivors, X-ray exposure to pediatric populations, and radiology staffs have indicated the long-term complications that are clearly visible in epidemiological studies.^13–16^ These studies show that low-dose radiation is associated with risks of carcinogenesis and functional impairment of the cardiac and circulatory systems.^17^ Several recent publications also have provided views against the use of LDRT due to radiation risk.^18,19^

In diagnostic imaging, the benefits outweigh the potential risks associated with low-dose radiation. The use of LDRT for Covid-19 patients has never been tested; thus, it is imperative that such decisions should be taken with great care. One could potentially evaluate current patients who are being imaged during the Covid-19 pandemic pre- and post-detection of pneumonia. The imaging dose could be categorized as low dose (0.01–0.015 Gy) and its efficacy can potentially be evaluated from a large pool of Covid-19 infected patients having CT. Additionally, the recent development of an ACE2 humanized mouse susceptible to SARS-CoV2 infection could also help in the conduct of preclinical studies to further support such trials.^20^ Currently, ongoing clinical trials are accruing patients and awaiting their results.^12^ Without preclinical data or well-conducted trials, it is premature to assume that LDRT is appropriate for Covid-19-induced pneumonia.

## Conclusion

Covid-19 seems mainly a lung disease with manifestation of pneumonia and possible rapid progress to heart and/or kidney failure and stroke with fatal consequences. Most patients get better without treatment as on average only a 6.9% death rate is reported with known cases, which indicates that various underlying factors are at play such as smoking, diabetes, cancer, and cardiac problems, all contributing to an immune-compromised condition. It is premature to advocate LDRT as a treatment for Covid-19-induced pneumonia unless a definite mechanism is found. We cannot use radiation for every possible disease, including Covid-19 pneumonia, unless there is a scientific study to prove its efficacy. Until that happens, let us wait on using LDRT for the treatment of Covid-19. In this context, one should pay attention to the very short-term probable gain, or the long-term problems associated with low-dose radiation that may haunt us in coming years.
